# The Role of DNA Methylation in Transcriptional Regulation of Pro-Nociceptive Genes in Rat Trigeminal Ganglia

**DOI:** 10.1177/2516865720938677

**Published:** 2020-09-10

**Authors:** Guang Bai, Holly Ross, Youping Zhang, KiSeok Lee, Jin Y Ro

**Affiliations:** Department of Neural and Pain Sciences, University of Maryland Dental School, Baltimore, MD, USA

**Keywords:** DNA methylation, inflammation, sensory ganglia, pain

## Abstract

Epigenetic modulation by DNA methylation is associated with aberrant gene expression in sensory neurons, which consequently leads to pathological pain responses. In this study, we sought to investigate whether peripheral inflammation alters global DNA methylation in trigeminal ganglia (TG) and results in abnormal expression of pro-nociceptive genes. Our results show that peripheral inflammation remotely reduced the level of global DNA methylation in rat TG with a concurrent reduction in *DNMT1* and *DNMT3a* expression. Using unbiased steps, we selected the following pro-nociceptive candidate genes that are potentially regulated by DNA methylation: *TRPV1, TRPA1, P2X3*, and *PIEZO2*. Inhibition of DNMT with 5-Aza-dC in dissociated TG cells produced dose-dependent upregulation of *TRPV1, TRPA1*, and *P2X3*. Systemic treatment of animals with 5-Aza-dC significantly increased the expression of *TRPV1, TRPA1*, and *PIEZO2* in TG. Furthermore, the overexpression of DNMT3a, as delivered by a lentiviral vector, significantly downregulated *TRPV1* and *PIEZO2* expression and also reliably decreased *TRPA1* and *P2X3* transcripts. MeDIP revealed that this overexpression also significantly enhanced methylation of CGIs associated with *TRPV1* and *TRPA1*. In addition, bisulfite sequencing data indicated that the CGI associated with *TRPA1* was methylated in a pattern catalyzed by DNMT3a. Taken together, our results show that all 4 pro-nociceptive genes are subject to epigenetic modulation via DNA methylation, likely via DNMT3a under inflammatory conditions. These findings provide the first evidence for the functional importance of DNA methylation as an epigenetic factor in the transcription of pro-nociceptive genes in TG that are implicated in pathological orofacial pain responses.

## Introduction

The study of epigenetic mechanisms underlying acute or persistent pain has been rapidly progressing in recent years.^1,2^ As a prototypical epigenetic factor, DNA methylation plays an important role in regulating gene expression, and alterations in DNA methylation are associated with a number of human diseases, including chronic pain conditions. Epigenetic modulation by DNA methylation is associated with hyperalgesic responses and aberrant gene expression in the central nervous system.^3,4^ The substantial amount of evidence accumulated thus far also indicates that alterations in DNA methylation in dorsal root ganglia (DRG) may play a critical role in the underlying mechanisms of many types of somatic chronic pain conditions.^5-8^

Gene-specific studies, genome-wide association studies, and the Pain Gene Database reveal that many genes that are involved in persistent pain contain CpG islands (CGIs) near their transcription start sites. These CGIs are highly sensitive to methylation, and some of these genes were found to either regulate or be regulated by DNA methylation.^9^ The blockade of DNA methyltransferases (DNMTs), key enzymes involved in DNA methylation, modulates pain responses under inflammatory and neuropathic pain conditions.^4,5,10^ More recent studies showed that nerve injury induces upregulation of DNMT3a in DRG and that a global blockade of this increase in DRG attenuates neuropathic pain.^7,8^ However, both the identity of pro-nociceptive genes that are subject to epigenetic modulation via alterations in DNA methylation in trigeminal ganglia (TG) and the role of DNMTs in pathological orofacial pain conditions are not clearly established.

We have previously shown that inflammation of the masseter muscle in rats upregulates transcription of many pro-nociceptive genes in TG that have been implicated in pathological orofacial pain responses.^11^ Among those genes, DNA methylation of the transient receptor potential ankyrin 1 (*TRPA1*) gene and, to a lesser extent, the transient receptor potential vanilloid 1 (*TRPV1*) gene in human blood sample is associated with pain sensitivity.^12-14^ However, it is not known whether the high expression of these genes in sensory ganglia is under the regulation of DNA methylation. Calcitonin gene-related peptide (*CGRP*), which partly mediates masseteric inflammatory mechanical hyperalgesia,^15^ is regulated by DNA methylation, but this was demonstrated in TG cultures primarily composed of glia.^16^ To the best of our knowledge, there have been no reports on how peripheral inflammation impacts the cellular components that regulate DNA methylation in TG. The objectives of this study are to investigate whether masseter inflammation alters global DNA methylation in TG and to identify potential cellular machineries and pro-nociceptive genes that are regulated by DNA methylation.

## Materials and methods

### Animals

Adult male Sprague Dawley rats (250-350 g; Harlan, Indianapolis, IN) were used. All animals were housed in a temperature-controlled room under a 12:12 light-dark cycle with access to food and water *ad libitum*. All procedures were conducted in accordance with the NIH Guide for the Care and Use of Laboratory Animals (Publication No. 80-23) and under a University of Maryland-approved Institutional Animal Care and Use Committee protocol.

### Muscle inflammation

Inflammation was induced by injecting 50 µl of 50% complete Freund’s adjuvant (CFA) in isotonic saline (Sigma, St. Louis, MO) into the mid-region of the masseter muscle unilaterally via a 27-gauge needle. Rats were briefly anesthetized with 3% isoflurane for the injection procedure. The characteristics of inflammation and behavioral responses following CFA injections in the rat masseter have been described previously.^17,18^

### Primary TG/DRG culture

All animals were euthanized by decapitation under isoflurane anesthesia. Both TG or all DRG from each animal were dissected out and dissociated by sequential digestion with 0.1% collagenase D in DMEM-F12 medium (with L-glutamine) at 37°C for 30 minutes, followed by additional digestion with 0.25% trypsin and 50 μg DNase in the same medium plus 0.02% EDTA at 37°C for 15 minutes. After trituration, cells were plated on laminin pre-coated 24-well plates and cultured in a 37°C incubator at 5% CO_2_ for 1 to 3 days. For some studies, dissociated TG or DRG cells were transduced by pseudo LV virions 5 hours after seeding on wells and harvested 3 days after culture seeding for RNA or gDNA extraction. Cells dissociated from a given animal were seeded on a set of wells to complete selective experiments as one biological replicate.

### Genomic DNA extraction

Genomic DNA (gDNA) was extracted from intact TG or dissociated TG cells according to the method described by Strauss^19^ and quantified by absorbance at 260 nm (A_260_) on a Nanodrop (ThermoFisher, Waltham, MA). Biochemical quality of gDNA was evaluated by A_260_/A_280_ (1.75~1.9) and A_260_/A_230_ (>1.0) ratios. Molecular integrity was determined by electrophoresis on 0.9% agarose gels, producing a single band approximately 40 kb. Qualified gDNA was saved in TE buffer (pH 8.0) at –20°C until use for methylation assay or bisulfite modification.

### Global DNA methylation assay

The concentration of 5-methylcytosine (5-mC) in gDNA was quantified using 5-mC DNA ELISA kit and was measured according to the manufacturer’s instructions (Zymo Research, Irvine, CA). Briefly, standard DNA used to establish the standard curve was prepared by mixing negative (unmethylated DNA at 100 ng/μl) and positive (completely methylated DNA at 100 ng/µl) controls at different proportions. Standard or sample gDNA of 100 ng each was mixed with 5-mC coating buffer up to 100 μl and denatured at 98°C for 5 minutes followed by quick transfer onto ice for 10 minutes. Denatured gDNA was coated onto 96-well plates by incubation at 37°C for 1 hour. Following 3 washes with 5-mC ELISA buffer, 200 μl of 5-mC ELISA buffer was added for another incubation at 37°C for 30 minutes. An antibody mixture consisting of anti-5-mC and secondary antibody in 5-mC ELISA buffer in the ratio of 1:2:2000 in 100 µl was added for incubation at 37°C for 1 hour. After washing, 100 µl HRP developer was added, and color development was achieved by incubation at room temperature for 1 hour. Then the absorbance at 450 nm was obtained from each reaction on a plate reader (BioTek Epoch 2, BioTek/Agilent, Santa Clara, CA) for the establishment of a standard curve and for the quantitative analyses of samples from the curve. The results were validated in triplicate for each standard and sample.

### Drug administration

For *in vivo* analysis, 5-Aza-dC (5-aza-2’-deoxycytidine) (Sigma-Aldrich, St. Louis, MO) was freshly dissolved in phosphate buffered solution (PBS), pH 7.4. 5-Aza-dC in 1 mg/100 µl of PBS was administered intraperitoneally (i.p.) to rats for 3 consecutive days, after which TG was dissected out for RNA or gDNA extraction. For *in vitro* experiments, TG cultures were treated with 3 different concentrations of 5-Aza-dC (2.5, 5, and 10 µg/ml) for 24 hours.

### Quantitative reverse transcriptase polymerase chain reaction (qRT-PCR)

TG was dissected from naïve rats and CFA-inflamed rats (3 days post CFA treatment) as well as from those receiving 5-Aza-dC injections. Total RNA was extracted from dissected TG or from dissociated TG cells in culture using an RNeasy kit (Qiagen Sciences, MD) followed by DNase treatment to remove possible contaminating gDNA. Reverse transcription was carried out using Superscript II kit (Invitrogen, CA) from 100 to 1000 ng of total RNA along with Oligo (dT) primer. Quantitative PCR analysis of cDNA equal to 20 ng RNA was performed on the Eppendorf Mastercycler EP Realplex 2.0. Primer sequences for *DNMT1, DNMT3a, DNMT3b, GADD45α, MBD4, TET1, TET2, TET3, TRPV1, TRPA1, P2X3*, and *PIEZO2* can be provided upon request. The cycling protocol used was 95°C for 5 minutes, followed by 40 cycles of 95°C for 15 seconds, 58°C for 15 seconds, and 68°C for 20 seconds. Relative level of the target mRNA was calculated by the comparative Ct method (ΔΔCt method) with normalization to beta actin mRNA between control and experimental groups.

### Design and validation of EGFP-DNMT3a chimeric construct

We cloned the full-length encoding sequence of the rat *DNMT3a* gene from a cDNA pool of rat TG and fused it to the c-terminus of enhanced green fluorescent protein (EGFP) in vector pEGFP-C1 (Clontech/Takara Bio, Mountain View, CA) to form the EGFP-DNMT3a chimera. The catalytic domain of DNMT3a is located at the c-terminus, and this chimera does not interfere with its activity.^20^ Because of the large size of this chimera (3.5 kb), we utilized a lentiviral (LV) vector (Addgene catalog #71237) and replaced the transgene expression cassette with the chimera. To increase the neuronal specificity of expression, we constructed a hybrid promoter consisting of the enhancer region of the human CMV early gene promoter and the basal promoter of the rat NMDAR1 gene, which contains an RE1 site for neuronal expression. A vector expressing only EGFP was constructed as a control. The fidelity of all cloned promoters, cDNA, and chimera was confirmed by DNA sequencing. Pseudo lenti virions were prepared using a third generation system and a standard protocol.^21^ We named this lenti vector LV-CMVeNR1-EGFP-DNMT3a. A pseudo lenti virion expressing only EGFP was also prepared and named LV-CMVeNR1-EGFP. We confirmed the activity and neuronal specificity of this hybrid promoter using a luciferase reporter gene assay in human HEK293 cells (non-neuronal) and rat PC12 cells (neuronal). Expression of the EGFP-DNMT3a chimera was then tested by transducing dissociated rat TG or DRG cells with the pseudo virions and examining the green fluorescence of EGFP. Transduced TG cultures exhibited green fluorescence in neurons 2 days post-infection. Compared to the cytosol of the EGFP control, the fluorescence of this chimera is largely limited to the nuclei, suggesting successful expression of DNMT3a and its nuclear localization feature.

### Analyses of methylated DNA by bisulfite (BS) sequencing and methylated DNA immunoprecipitation (MeDIP)

Analysis of methylated DNA by BS sequencing was completed as described previously.^22^ Briefly, BS conversion was performed for 100 ng purified gDNA with EZ DNA Methylation Gold kit (Zymo Research) according to the manufacturer’s instructions. *TRPA1* CGI was amplified by PCR with primers whose sequence may be provided upon request. The PCR amplicon was subcloned into the pGEM-T vector (Promega, Madison, WI) and sequenced by Genewiz service (South Plainfield, NJ).

MeDIP was adapted from a published study.^23^ Briefly, 300 ng of gDNA was sonicated to 200 to 1000 bp fragments using the Covaris S2 Ultrasonicator (intensity 5, 10% duty cycle, 200 cycles per burst, and 2 × 30 seconds with 30 seconds intervals). DNA fragmentation was verified on an Agilent BioAnalyzer gel. 100 ng of denatured fragmented DNA was immunoprecipitated with 0.2 µg of monoclonal 5 mC antibody (33D3, Abcam, Cambridge, MA), and then 1 µg rabbit anti-mouse IgG was added prior to the addition of magnetic Protein A/G beads (Invitrogen/ThermoFisher, Waltham, MA). After washing as described in the referenced protocol, precipitated DNA was subjected to Protease K digestion and phenol-chloroform extraction. Extracted DNA was used in qPCR with SYBR Green/ROX reagent and 10 µM primers designed for the TRPA1 gene or TRPV1 gene (primer sequences available upon request). The thermocycler program included a 95°C step for 4 minutes, followed by 50 cycles of 95°C for 15 seconds and Tm for 60 seconds. Non-precipitated DNA fragments were diluted accordingly and utilized as an input control, and the relative level of enrichment was calculated using the ΔΔCt method.

### Statistical analysis

Statistical comparisons of 2 independent groups were made with Student’s *t*-test. For multiple group comparisons, one-way analysis of variance (ANOVA) or Kruskal–Wallis one-way ANOVA on ranks was performed depending on the outcome of the normality test, followed by Dunnett’s post hoc test. All statistical analyses were conducted with GraphPad software. Data analyzed with a parametric test are presented as mean ± SE, and data analyzed by a non-parametric test are presented as median with interquartile range in box plots. Differences were considered significant at *P* < .05. The sample size for each experiment is shown in the graph or described in the figure legend.

## Results

### Peripheral inflammation alters global DNA methylation and DNMT expression in TG

**Figure 1.**
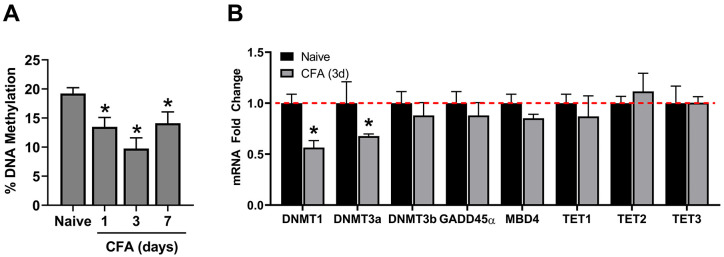
Effects of inflammation on DNA methylation and on enzymes that regulate DNA methylation in TG. (A) The extent of gDNA methylation was compared between naïve and CFA-treated rats (n = 5/group). (B) mRNA levels for *DNMT1, DNMT3a, DNMT3b, GADD45α, MBD4, TET1, TET2*, and *TET3* detected by qRT-PCR were compared between naïve and CFA-treated rats on day 3 (CFA, 3d) (n = 4/group).*P < .05.

We first examined whether CFA-induced inflammation of the masseter muscle is associated with alterations in global DNA methylation in TG and whether the changes in DNA methylation were reversible. Masseter inflammation resulted in a significant decrease in the percent DNA methylation in TG from 1 day after CFA injection. The decrease was greatest on day 3 and reversed toward baseline by day 7 at a level comparable to day 1 (Figure 1A).

DNA is methylated by 3 active DNMTs in mammalian cells, that is, DNMT1, DNMT3a, and DNMT3b.^24^ We predicted that inflammation-induced reduction in the global methylation level in TG would be accompanied primarily by a decrease in the level of DNMT expression. We examined the levels of DNMT transcripts in TG of naïve and CFA-inflamed rats 3 days after CFA treatment in light of the fact that the greatest reduction in global DNA methylation was at this time point. Our results indicated that the expression of *DNMT1* and *DNMT3a*, but not *DNMT3b*, in TG of inflamed rats was significantly reduced (Figure 1B).

It has been known that the level of DNA methylation in a given cell is regulated not only by DNMTs, but also by enzymes that actively demethylate DNA. Although the molecular mechanism underlying active DNA demethylation is relatively poorly understood, growth arrest and DNA-damage-inducible protein 45 alpha (GADD45α), a nuclear protein involved in maintenance of genomic stability, is known to demethylate DNA via nucleotide excision repair to erase 5-mC.^25^ Also, the mammalian DNA glycosylase-methyl-CpG binding domain protein 4 (MBD4) has been implicated in playing a role in active DNA demethylation by enzymatic removal of the methyl group from 5-mC.^26^ Our data indicated that neither *GADD45α* nor *MBD4* expression at the mRNA level was significantly altered in TG of animals treated by CFA (Figure 1B). More recently, ten-eleven translocation (TET) proteins have been discovered as 5-mC oxidases that actively demethylate DNA via the iterative oxidation of methyl groups.^27^ However, none of the genes encoding enzymes in the TET family showed significant changes following the CFA treatment (Figure 1B). These results suggest that DNMT1 and DNMT3a likely play a primary role in global DNA demethylation in TG during masseter inflammation.

### Selection of target genes modulated by DNA methylation following masseter inflammation

Changes in global DNA methylation in TG may constitute composite responses of multiple genes. Here we hypothesized that pro-nociceptive genes may be upregulated at the transcriptional level following CFA-induced alteration of DNA methylation, thereby allowing their participation in the development of inflammatory mechanical hyperalgesia. To test this hypothesis, we used the following steps to select candidate molecules in a relatively unbiased manner: (1) identify pain-related genes that are significantly upregulated in TG following masseter inflammation from a genome wide assay study of our RNAseq analysis,^11^ (2) conduct a literature search to identify molecules that have been demonstrated to contribute to inflammatory mechanical hyperalgesia arising from the masseter muscle, and (3) conduct genome browser searches to examine whether CGIs are predicted in genes that satisfy steps 1 and 2.

Previously, in RNAseq analysis, we selected 2320 candidate genes that the literature revealed to be relevant to nociceptors, implicated in pain mechanisms, enriched in small—to medium-sized sensory neurons, and enriched in TRPV1-lineage nociceptors.^11^ Among these candidate genes, 622 genes showed differential expression in TG following masseter inflammation. Upon further literature search, we identified following pro-nociceptive genes that show a significant transcriptional increase during inflammation-induced mechanical hyperalgesia: *TRPV1* (fold change 1.43, *P* < .001 vs non-inflamed animals), *TRPA1* (fold change 1.21, *P* < .001), and *P2X3* (fold change 1.27, *P* < .001).^28-32^ *CGRP* has been implicated in masseter inflammatory mechanical hyperalgesia,^15^ but the fold change did not reach statistical significance in our RNAseq analysis. *PIEZO2*, which has been implicated in mechanical pain under pathological conditions,^33,34^ showed significant upregulation (fold change 1.42, *P* < .05) in TG.

### Effects of 5-Aza-dC on the expression of target genes in TG

**Figure 2.**
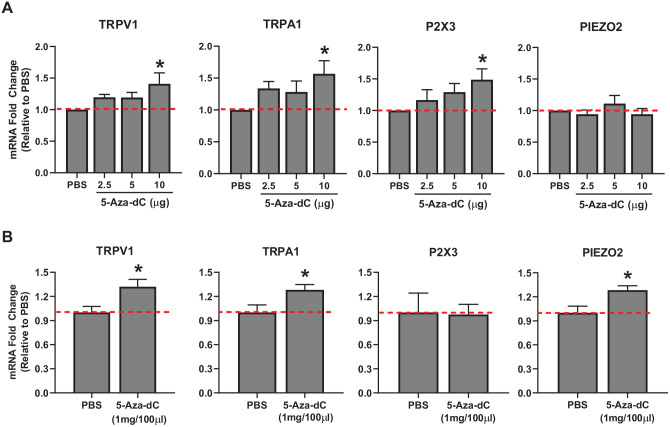
Effects of 5-Aza-dC on mRNA level of pro-nociceptive genes in TG. (A) TG cultures treated with vehicle (PBS) or 1 of the 3 doses of 5-Aza-dC for 24 hours. N = 5 to 7. qRT-PCR was conducted as described in methods. *P < .05 compared to the vehicle condition analyzed with one-way ANOVA. (B) 5-Aza-dC or its vehicle (PBS) was administered (i.p.) for 3 consecutive days after which TG was extracted and analyzed. Each group consisted of 5 to 7 rats. *P < .05 between the 2 groups analyzed with Student’s *t*-test.

In order to demonstrate that the transcription of these candidate genes is actually modified by DNA methylation, we pharmacologically inhibited DNA methylation and assessed the expression levels of these genes in TG under both in vitro and in vivo conditions. Treatment of TG primary cultures with 5-Aza-dC, a DNMT inhibitor, dose-dependently increased the expression of *TRPV1, TRPA1*, and *P2X3*, but not *PIEZO2* (Figure 2A). Furthermore, systemic treatment of 5-Aza-dC (i.p.) for 3 days in intact animals significantly upregulated the expression of *TRPV1, TRPA1*, and *PIEZO2*, but not *P2X3* in TG (Figure 2B). These data suggest that alterations in *TRPV1* and *TRPA1* expression are reliably observed following the inhibition of DNA methylation, and, though less reliably observed, the expression of *P2X3* and *PIEZO2* is also subject to modulation by DNA methylation.

### Effects of DNMT overexpression in TG

**Figure 3.**
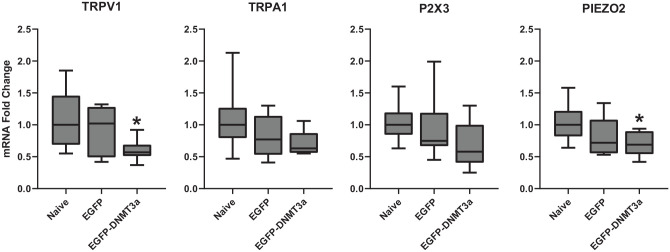
Effects of LV-mediated DNMT3a overexpression on mRNA of pro-nociceptive genes in TG. mRNA fold changes in TG cultures treated with pseudo-viruses (LV-CMVeNR1-EGFP-DNMT3a for EGFP-DNMT3a or LV-CMVeNR1-EGFP for EGFP) were compared to those of untreated TG cultures (naïve). Median values with interquartile range in box plots are presented. N = 9. *P < .05 compared to naive condition.

Generally, DNMT3a is implicated in *de novo* DNA methylation while DNMT1 maintains constitutive DNA methylation.^9^ Interestingly, the role of DNMT3a in persistent pain remains controversial.^5,7,8,35,36^ All of these factors prompted us to test whether DNMT3a-produced DNA methylation is involved in alteration of the 4 candidate genes above. Considering that 5-Aza-dC inhibits all known DNMTs, and DNMT3a-specific or isoform-specific inhibitors are still under development,^37,38^ we addressed this question by overexpressing DNMT3a. We transduced TG cultures with an LV vector (LV-CMVeNR1-EGFP-DNMT3a) expressing chimera of EGFP and full-length DNMT3a. LV pseudo virus expressing EGFP alone (LV-CMVeNR1-EGFP) was used as a control. The LV-induced changes in mRNA levels of target genes were compared to those from untreated naïve cultures. As expected, LV-CMVeNR1-EGFP-DNMT3a treatment led to a consistent reduction in mRNA expression for all 4 genes, that is, *TRPV1, TRPA1, PIEZO2*, and *P2X3* whereas LV-CMVeNR1-EGFP treatment had no significant impact on these genes in comparison to those in naive TG cultures (Figure 3). LV-CMVeNR1-EGFP-DNMT3a significantly down-regulated *TRPV1* and *PIEZO2* expression, but also reliably decreased *TRPA1* and *P2X3* transcripts. Although the decrease of *TRPA1* and *P2X3* expression levels did not reach statistical significance, the LV-CMVeNR1-EGFP-DNMT3a treatment resulted in the reduction of target gene mRNAs more frequently than it resulted in an increase of the same mRNAs. As shown in Table 1, LV-CMVeNR1-EGFP-DNMT3a transduced cells yielded a much higher incidence of decreased mRNA levels for the 4 tested genes (78%~100% of assays) than that yielded by LV-CMVeNR1-EGFP transduced cells (67% of assays or less). In fact, every assay of LV-CMVeNR1-EGFP-DNMT3a transduced cells resulted in downregulation of *TRPV1, TRPA1*, and *PIEZO2*. The low frequency of P2X3 assays (less than a quarter) showing higher expression after LV-CMVeNR1-EGFP-DNMT3a treatment than the average naïve control may be due to technical variation caused by the low transduction rate of primary cultures containing numerous cell types. This kind of variation may also explain the lack of statistical significance of the reduction of measured TRPA1 mRNA levels. Therefore, we believe that overexpressed EGFP-DNMT3a downregulates *TRPV1, TRPA1, P2X3*, and *PIEZO2*.

**Table 1.** Percentage of assays showing fluctuation of target gene levels in either direction in TG cells transduced by indicated lentiviral vector.

LV-CMVeNR1-	Target mRNA and fluctuation							
	TRPV1		TRPA1		P2X3		PIEZO2	
	Up	Down	Up	Down	Up	Down	Up	Down
EGFP-DNMT3a	0	100	0	100	22	78	0	100
EGFP	56	44	44	56	33	67	33	67

DNA methylation of cytosine residues mostly occurs in CpG dinucleotides, which are abundant in CGI.^8^ Searching the UCSC Genome Browser (genome.UCSC.edu), we found 1 and 3 CGIs associated with *P2X3* and *PIEZO2*, respectively. We further examined CGI for TRPA1 and *TRPV1* using MethPrimer software (urogene.org/methprimer), the most popular software for predicting CGIs with an algorithm of relatively lower stringency. Our search results showed that the rat TRPA1 gene (ENSRNOG00000007354, Ensembl.org) contains 1 CGI (139 bp) starting 25 bp upstream of its open reading frame, and the rat TRPV1 gene (ENSRNOG00000019486, Ensembl.org) has 1 CGI in intron 4 (243 to 348 of intron 4 sequence) and 1 in intron 5 (190 to 315 of intron 5 sequence).

**Figure 4.**
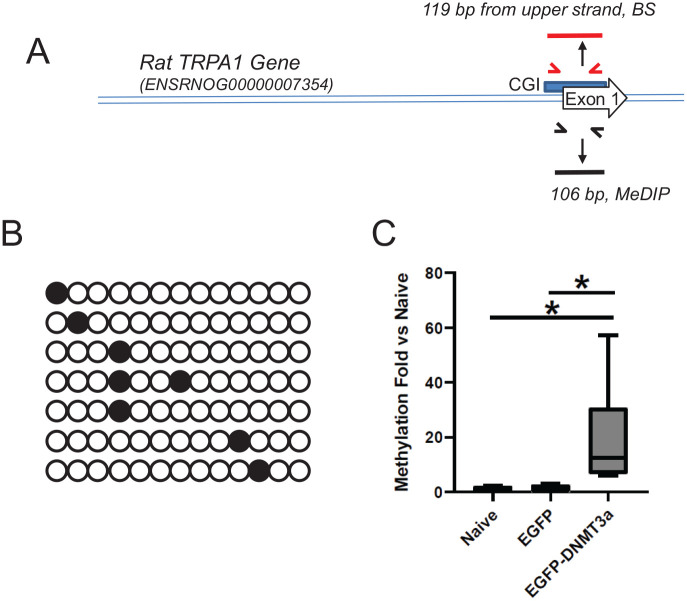
Methylation analyses of the TRPA1 gene. (A) Schematic presentation of PCR primers for *TRPA1* MeDIP and bisulfite-modified *TRPA1* CGI. Primers were designed based on sequences of the rat TRPA1 gene. (B) Representative methylated clones of BS sequencing of the *TRPA1* CGI. CpGs are presented as circles aligned in a row for each individual clone. Open circles indicate unmethylated CpGs, and solid circles indicate methylated CpGs. (C) MeDIP results of the *TRPA1* CGI. Data are presented in the same format as in Figure 3. Experiments were performed as described in methods.N = 7~8. *P < .05 compared to naive condition.

To test the possibility of DNA methylation, we conducted BS modification of TG genomic DNA (gDNA) and cloned a 119 bp portion of the *TRPA1* CGI from a PCR amplicon (Figure 4A). Sequencing of clones revealed that this CGI can indeed be methylated and, importantly, the methylation pattern matches those produced by DNMT3a, that is, randomly on the CpG sites of each DNA strand (Figure 4B).^39^ However, the methylation rate of analyzed clones, each of which may maximally represent a single genome, is very low (~7%), preventing us from efficiently analyzing the methylation induced by DNMT3a. Although only a very limited of clones were analyzed, this low methylation rate is likely due to the fact that only a few neurons in the mixed cell population of TG determine changes in target gene expression.

**Figure 5.**
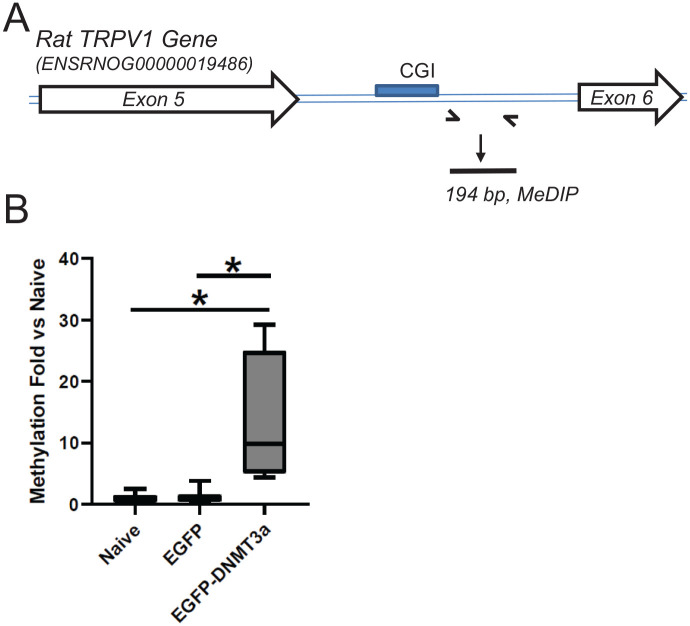
Methylation analyses of the TRPV1 gene. (A) Schematic presentation of PCR primers for *TRPA1* MeDIP. Primers were designed based on sequences of the rat TRPV1 gene. (B) MeDIP results of the *TRPV1* CGI. Others are the same as Figure 4.

To further test whether this methylation is indeed catalyzed by DNMT3a in TG neurons and to avoid the bias of individual clones in BS sequencing, we transduced dissociated DRG cells with LV-CMVeNR1-EGFP-DNMT3a or LV-CMVeNR1-EGFP, used MeDIP to enrich methylated DNA fragments (500-1000 bp) from gDNA of transduced cells, and detected CGIs of *TRPV1* (in intron 5) and of *TRPA1* using quantitative PCR (Figures 4A and 5A). DRG cells transduced by LV-CMVeNR1-EGFP-DNMT3a demonstrated a significant increase in methylated DNA in comparison to naïve cells, while cells transduced by LV-CMVeNR1-EGFP were barely impacted (Figures 4C and 5B). These results demonstrated that, along with their adjacent sequences on both strands, CGIs identified from *TRPV1* and *TRPA1* are methylated by DNMT3a.

## Discussion

The main findings of this study are: (1) Inflammation in orofacial muscle tissue leads to both global reductions in DNA methylation and to downregulation of *DNMT1* and *DNMT3a* expression in TG. (2) Many pro-nociceptive genes are subject to transcriptional regulation via DNA methylation. (3) DNMT3a is a critical enzyme mediating these alterations in DNA methylation. We demonstrated that the expression of at least 4 pro-nociceptive genes, that is, *TRPV1, TRPA1, P2X3*, and *PIEZO2*, are regulated by this epigenetic mechanism. (4) DNMT3a at least acts on CGIs associated with *TRPV1* and *TRPA1*. These findings are discussed in terms of primary afferent processing of pathological pain responses that arise from orofacial structures in the presence of inflammation.

### Peripheral inflammation alters global methylation level in TG

Global methylation, which represents the overall state of the DNA methylation of a given tissue, has long been associated with cancer pathogenesis and progression.^40-42^ Relatively little information on changes in global methylation along nociceptive pathways is available. Nerve injury induces chronic, but reversible, reductions in global methylation in the mouse prefrontal cortex, which correlate with mechanical and thermal sensitivity in neuropathic mice.^4^ A more recent study showed that nerve injury induces persistent reprogramming of global methylation in DRG and that DNA hypomethylation occurs during the chronic phase of neuropathic pain.^43^ In addition, the same study demonstrated that pharmacological or dietetic induction of global DNA hypomethylation in DRG causes long-lasting hypersensitivity. These observations provide evidence that the status of global methylation in DRG is predictive of pain phenotypes under nerve injury conditions. However, functional correlations of global DNA methylation with pain responses appear to be tissue-specific since neither nerve injury^43^ nor tissue injury^44^ caused global DNA hypomethylation at the spinal cord level.

Our data showed time-dependent changes in global DNA methylation in TG following CFA-induced inflammation of the masseter muscle. Although the assessment was made transiently for 7 days following the inflammation, the time course of hypomethylation correlates well with the time course of inflammation-induced pain responses.^45^ It is acknowledged that changes in global methylation are composite responses from a multitude of genes, including those that do not have any direct relevance to pain processing. There is a significant change in the TG transcriptome, which involves a substantial number of pain-related genes, 3 days following masseter inflammation, the time point at which the reduction of DNA methylation was the greatest.^11^

Since global DNA methylation can be correlated with affected behaviors and physiology at a tissue-specific level, it would be interesting to investigate whether restoring the DNA hypomethylation in TG would attenuate inflammatory pain. Positive outcomes would provide further support for targeting global DNA methylation as a novel therapeutic approach for treating chronic and pathological pain conditions.

### Peripheral inflammation leads to downregulation of DNMT1 and DNMT3a in TG

DNA methylation in mammalian genomes is dynamically regulated by complex interactions of multiple enzymes that either methylate or demethylate cytosine, a highly conserved epigenetic modification site in DNA.^27^ Among those enzymes, the roles of DNMT1,^6^ DNMT3a,^7,8,35^ DNMT3b,^46^ MBDs,^10,47^ GADD45α,^48^ and, most recently, TET^49^ in pain mechanisms have been explored. Our data show that peripheral inflammation resulted in a significant downregulation of *DNMT1* and *DNMT3a* expression in TG, without altering the expression levels of *GADD45*α, *MBD4*, and *TETs*, suggesting that DNMT1 and DNMT3a play a leading role in the CFA-induced decrease in global methylation in TG.

There are, however, conflicting data on inflammation-induced changes in DNMT expression levels in sensory ganglia. In an instance, CFA-induced inflammation in the hindpaw led to a significant reduction of *DNMT3b*, but not *DNMT3a*, expression in DRG.^46^ In other studies, peripheral inflammation did not alter either *DNMT3a* or *DNMT3b* expression in DRG.^5,8^ Nerve injury leads to increased expression of *DNMT1* and *DNMT3a* in DRG^7,8,35^ whereas skin incision does not alter the expression of any of DNMTs in DRG.^44^ Interestingly, the changes in DNMT expression levels in DRG are not always accompanied by the same changes in the injured tissue or in the spinal cord.^3,44^ These observations suggest that changes in DNMT expression in sensory ganglia are injury- and tissue-specific. Thus, the reduction of *DNMT1* and *DNMT3a* observed in our study may represent specific changes in DNMTs that occur in TG following inflammation of the craniofacial muscle tissue. More notably, our data provide support that reduced DNMT3a expression relieves the suppression of pronociceptive genes for ion channels in inflammatory pain.

A series of studies from a lab showed that restoring global DNMT expression levels within a tissue following a nerve injury is sufficient to alter pain responses,^6-8^ although the functional role of DNMT3a in mouse sensory neurons in persistent pain has recently been challenged.^36^ Given that DNMTs could alter a multitude of genes that are either pro-nociceptive or anti-nociceptive, that DNMT expression levels are sensitive to changing tissue environments, and that DNMT is only one of many families enzymes involved in gene transcription, additional studies are warranted to confirm whether global changes in DNMT expression levels bear any functional relevance in pain modulation.

### DNMT3a is involved in transcriptional regulation of pro-nociceptive genes in TG

DNMT-catalyzed methylation of CpG dinucleotides typically results in the repression of gene transcription. Under neuropathic and chemotherapy-induced nerve injury conditions with increased DNMT3a levels in DRG, transcription of anti-nociceptive genes in DRG, such as mu opioid receptor and various types of voltage-gated potassium channels, are repressed.^7,8,35^ A significant downregulation of DNMT3b has been associated with an increased expression of pro-nociceptive chemokine receptor *CXCR4* in DRG.^46^ Here we provided additional evidence that DNMT3a is involved in the transcriptional regulation of key pro-nociceptive genes in TG. We used a relatively unbiased and rigorous protocol for identifying candidate genes likely to be modulated by DNA methylation in craniofacial inflammatory pain: (1) RNA sequencing of TG to identify pain-related genes upregulated following masseter inflammation, (2) literature search to identify candidates that contribute to mechanical hyperalgesia in craniofacial muscles, (3) genome browser search to confirm CpG islands in genes from 1 and 2, and (4) assessment of genes that show transcriptional modulation by 5-Aza-dC.

Our *in vitro* and *in vivo* experiments with 5-Aza-dC strongly associate DNMT3a with *TRPV1* and *TRPA1* expression, and less reliably, but significantly, with *P2X3* and *PIEZO2* expression in TG. The role of DNMT3a is further supported by our data showing that overexpression of DNMT3a results in significant downregulation of the candidate genes and an increase in DNA methylation of identified CGIs associated with *TRPV1* and *TRPA1* genes. Of course, we recognized that CpGs outside these tested CGIs may also be the target of DNMT3a. Based on these collective observations, we can propose that DNMT3a plays a critical role by maintaining DNA methylation levels of these, and possibly other, pro-nociceptive genes in our model of craniofacial muscle pain.

The reduction of DNMT3a level under pathological pain condition can result in aberrant expression of multiple pro-nociceptive genes, ultimately leading to pathological pain responses. Therefore, it is possible that both pharmacological and gene expression approaches on DNMTs could non-specifically affect DNA methylation of a large number of genes that are both pro-nociceptive and anti-nociceptive, and the contribution of individual genes in pain responses cannot be directly assessed. Studies that modulate global DNMT activity pharmacologically or through forced DNMT3a expression in a target tissue only allow for the establishment of a correlation between DNA methylation and gene regulation. In this study, we examined 4 pro-nociceptive genes among those that can be targeted by such manipulation. Although those genes have already been amply studied in pain mechanisms, understanding how each individual gene is epigenetically regulated by DNA methylation at the transcription level under inflammatory conditions could shed additional light on nociceptor mechanisms underlying the development and maintenance of craniofacial muscle pain conditions. We certainly acknowledge that these genes by no means constitute the exclusive set of genes modulated by DNA methylation in TG under masseter inflammatory conditions. It will be interesting to examine whether specific manipulation of DNA methylation of a single selected gene in TG is able to alter pain responses.
